# Real-Time PCR Assay for the Identification of the Brown Marmorated Stink Bug (*Halyomorpha halys*)

**DOI:** 10.3389/fmolb.2016.00005

**Published:** 2016-02-26

**Authors:** Manpreet K. Dhami, Melissa Dsouza, David W. Waite, Diane Anderson, Dongmei Li

**Affiliations:** ^1^Plant Health and Environment Laboratory, Ministry for Primary IndustriesAuckland, New Zealand; ^2^Department of Biology, Stanford UniversityStanford, CA, USA; ^3^Department of Ecology and Evolution, University of ChicagoChicago, IL, USA

**Keywords:** qPCR, invasive species, diagnostic test, Pentatomidae, biosecurity

## Abstract

The brown marmorated stink bug, *Halyomorpha halys* (Hemiptera: Pentatomidae), is a gregarious crop pest that has rapidly spread across the world in the last two decades. It is an excellent hitchhiker species, especially as an over-wintering adult. During this period it is often associated with non-biological commodities such as shipping containers and machinery that travel long distances. Inadequate identification keys and similarity to common species has assisted its spread across Europe, while accurate identification from immature stages or eggs is not possible. We developed a real-time TaqMan PCR assay for the accurate and sensitive detection of the brown marmorated stink bug from all life stages. The assay performance against required diagnostic criterion and within a quarantine framework are described.

## Introduction

Effective management of a pest relies heavily on is its rapid and accurate identification. Such techniques are often not available for newly emerging pests, for example, the brown marmorated stink bug (BMSB), *Halyomorpha halys* (Hemiptera: Pentatomidae). A plant pest affecting ornamentals, crops, weeds and even forest trees, the BMSB poses an economic threat to a wide variety of fruit crops including apples, pears, persimmon and peaches across China, Japan, Korea, and the USA (Funayama, 2004; Yu and Zhang, 2007; Nielsen and Hamilton, 2009; Son et al., 2009). Brown marmorated stink bug is an insect vector of the phytoplasma disease of *Paulownia tomentosa* (empress tree or foxglove tree) in Asia and is a suspected vector of other phytoplasmas (Jones and Lambdin, 2009). Additionally, it is a public nuisance as adult stink bugs aggregate in large numbers on the outside of buildings in late autumn, eventually entering them to overwinter (Hamilton, 2009), and discharging an unpleasant and long lasting odor on disturbance.

The BMSB is native to China, Japan, Korea and Taiwan but has rapidly spread around the world. It has recently invaded North America causing significant economic losses in the fruit industry as well as exerting pressure to soybean crops within 8 years of detection (Nielsen and Hamilton, 2009; Nielsen et al., 2011). It is now found across eastern and western United States, and Alberta, British Columbia, Ontario and Quebec in Canada (Jones and Lambdin, 2009). A similar spread of BMSB has also been reported across mainland Europe, with the first interception in Switzerland in 2004 (Wermelinger et al., 2008), and subsequent records from Germany (2011) (Heckmann, 2012), France (2012) (Callot and Brua, 2013), Hungary (2013) (Vétek et al., 2014), Greece (2011) (Milonas and Partsinevelos, 2014) and Italy (2013) (Maistrello et al., 2014). Between 2005 and October 2015, it has been intercepted 83 times at the New Zealand (Harris, 2010; IDC PHEL, 2013). It could likely enter New Zealand as a hitchhiker on non-biological items, as well as biological items such as fresh produce, cut flowers or nursery stock. New Zealand falls under high risk of establishment for this species based on its current global distribution. Recent modeling by Zhu et al. (2012) identifies New Zealand as having high climate suitability for the establishment of BMSB. In part due to the high interception rate, as well as its ability to spread, it is important to develop rapid and sensitive identification techniques that are independent of the target organism's life stage.

The BMSB belongs to the subfamily Pentatominae that comprises 54 described genera and hundreds of species. The genus *Halyomorpha* has 16 described species from Sub-Saharan Africa alone (Robertson, 2009). Little is known of this genus from the rest of the world. The only two species for which cytochrome c oxidase sequences are available on online databases such as GenBank and BOLD are *Halyomorpha halys* and *H. picus*. A total of 23 haplotypes based on the COI sequences of BMSB have been described, suggesting high variability within the species (Xu et al., 2013). Similarly, 19 haplotypes based on the 12S ribosomal RNA gene sequence and 18 haplotypes based on the COII gene sequence were found in the invaded regions of Asia (Xu et al., 2013). Of these only two 12S haplotypes and one COII haplotype were found in the invaded regions of USA (Xu et al., 2013). It is possible that a more conserved region such as the 28S or COI would be more suitable for the developmenmt of a qPCR assay. However, only few sequences of these genes from closely related organisms were available in the databases.

This study provides a species-specific and sensitive TaqMan real-time PCR assay for the identification of BMSB. The target gene for the development of this assay is cytochrome oxidase I (COI), since it is well described for most insect species including Pentatomidae. The developed assay as well as performance criteria such as analytical and diagnostic specificity, sensitivity, repeatability, reproducibility and blind panel testing, are reported here.

## Materials and methods

### Sample collection and identification

Brown marmorated stink bug samples intercepted at the New Zealand borders largely constituted the tested samples. These intercepted samples originated from a wide range of locations, including China, Japan and USA. Few Pentatomidae are present in New Zealand, and none from the genus *Halyomorpha. Fourteen* species of Pentatomidae and close relatives from New Zealand were obtained from the Plant Health and Environment Laboratory (PHEL, Auckland New Zealand) collections, as well as from Canada, Japan, USA and China. All samples were identified morphologically by entomologists at PHEL, and identifications were confirmed by sequencing their barcode region, as discussed below. Voucher specimens for each organism are held in the PHEL ethanol and voucher collections and are available upon request. All organisms imported into New Zealand were in accordance to the Import Health Standard, Section 22 of the Biosecurity Act 1993. Ethics approval was not required as insects are not classified as animals for the purposes of the Animal Welfare Act, 1999, New Zealand Legislation.

### DNA extraction, PCR amplification and sequencing

Total gDNA was extracted using DNeasy Blood and Tissue kit (Qiagen, CA, USA). A single leg was physically disrupted and finely chopped with sterile scissors. DNA quantification was conducted using the MultiSkan GO DNA quantification system with a μDrop^*^ plate (Thermo Fisher Scientific, MA, USA).

All the samples used in the assay development were identified via conventional PCR amplification and sequencing of the COI gene region, using the COI primer pair, LCO1490 and HCO2198 (Folmer et al., 1994). Each 20 μL reaction consisted of Red N'Amp master mix (Sigma-Aldrich Co., MO, USA), 250 nM of each primer, 0.04 μg/μL Bovine Serum Albumin (BSA) (Sigma-Aldrich Co.), 2–5 ng of DNA template and PCR-grade water. PCR cycling with a 2 min initial denaturation at 94°C, was followed by 30 cycles of 15 s at 94°C, 30 s at 52°C and 45 s at 72°C, followed by a final extension step of 7 min at 72°C. The approximately 700 bp amplicons were visualized via gel electrophoreses as described in Dhami and Kumarasinghe (2014). PCR products were sequenced bi-directionally using the amplification primers, by EcoGene (Auckland, New Zealand). All sequences were edited using Geneious version 7.1.5 (Kearse et al., 2012). Sequences were compared to the NCBI GenBank nucleotide or BOLD (Ratnasingham and Hebert, 2007) databases using BLAST (Altschul et al., 1990) to confirm morphological identification. These sequences were used to guide the development of the assay.

### Real-time PCR assay design and optimization

COI gene sequences of Pentatomidae, especially of the closely related Pentatominae, were obtained from samples described above, and were also downloaded from GenBank and BOLD sequence databases (Table S1). A unique region of the BMSB sequence was selected from the alignment and Beacon Designer 8.01 (Premier Biosoft, Palo Alto, CA, USA) was used to design the assay. Multiple primer and probe options were generated, and the best assay was determined as the primer and probe combination that maximized mismatches with closely related sequences.

A real-time PCR protocol was developed using BMSB positive control samples as well as several non-target species samples as negative controls. The designed primers and probes were tested on a CFX96 Touch Real-time platform (BioRad, Hercules, CA, USA). Assay was optimized using gradient PCR for annealing temperature (60–65°C), primer concentration (200 and 400 nM), and probe concentration (200 and 400 nM) using the SsoAdvanced Probes Mix (BioRad, Hercules, CA). For each reaction, 96-well clear bottom plates were used and all samples, standards and controls were run in duplicate wells. Fluorescence data was acquired to the FAM channel at the end of each cycle. The amplification curves were visualized using the CFX Manager Software v. 3.0 (BioRad).

### Analytical and diagnostic specificity of the assay

We included a total of 63 voucher specimens in this assay of which 13 were target and the remaining non-target species. Percentage of samples of known identity of the target that return a positive result contribute to the analytical specificity and non-target samples that return a negative result contribute to the diagnostic specificity of the assay. All the samples used for assay validation were identified prior to testing by conventional PCR and sequencing.

### Analytical sensitivity, amplification efficiency, repeatability and reproducibility of the assay

As described in Dhami et al. (2015), a 714 bp template COI gene region was used to prepare plasmid standards and diluted to a series of known copy number. Amplicon cloning was performed using the TOPO TA vector Cloning kit (Invitrogen, Carlsbad, CA, USA) as per the manufacturer's instructions, for two biological samples PQ20 and PQ23. For each sample, two clones, containing the correct insert were selected for preparing standards.

Plasmid DNA was extracted, quantified and digested to linearize it, as described previously (Dhami and Kumarasinghe, 2014; Dhami et al., 2015). Copy number was calculated as described previously (Dhami et al., 2015).

A dilution series of 10^9^–10 copies of linearized plasmid/reaction, run in triplicate, was used to determine the analytical sensitivity of the developed assay. Amplification curves were fitted using linear regression in the R environment (R Core Team, 2013). The qpcR package was used to calculate the efficiency of the reaction using the formula, *E* = (10−1∕*slope*) (Ritz and Spiess, 2008), following conversion to percentage efficiency, and the fit of the slope was recorded as *r*^2^. Standard curve data were used to calculate linear dynamic range and limit of detection (Bustin et al., 2009).

Intra-run variation or repeatability and inter-run variation or reproducibility were calculated by *Cq* mean standard deviation and percent coefficient of variation (%CV) for this non-quantitative assay. We tested five samples in triplicate, in two separate runs and calculated the %CV for each sample, to estimate repeatability. We compared the data from the two runs to estimate reproducibility.

### Blind panel validation of the assay

A total of 24 insect samples were used for blind panel testing. These samples were obtained from 12 different Pentatomidae species, representing various life stages and body parts, largely interceptions at the New Zealand borders. DNA extraction and quantification was performed as previously described. Samples were tested in duplicate and positive controls and no template controls were included. All samples were tested simultaneously with an internal positive control using a commercial 18S realtime PCR kit (Applied Biosystems, CA, USA). These experiments were essentially a simulation of the New Zealand quarantine framework, in which the developed assay was applied.

## Results

### BMSB assay: identification via taqman real-time PCR

Primer pair, Hhal1dF (5′–GAG GAT TCG GTA ATT GAT TA–3′) and Hhal1dR2 (5′–GTG AGA TAT TAC TTG ATA AGG–3′), amplify a 186 bp of the target sequence. Probe Hhal4P (5′-CTG ATA TAG CCT TCC CAC GAT TAA AT-3′), was selected after rigorous *in silico* testing for binding against the sequence alignments using Geneious. The final probe was synthesized with 5′FAM and 3′BHQ-1 modifications by Biosearch Technologies (CA, USA). Based on gradient PCR results, final cycling conditions were selected as follows: 95°C for 2 min, followed by 40 cycles of denaturing (95°C for 15 s) and annealing/extension (62°C for 45 s), followed by a plate read step. Reactions contained 400 nM of each primer with 200 nM of probe. Approximately, 10–30 ng/uL of template DNA was used for the PCR assay. The assay cut-off was set at 35 cycles to reduce possible non-specific binding.

### Analytical and diagnostic specificity of the assay

Of the 63 samples tested, 13 were target and the remaining non-target species. All the target BMSB samples amplified within the *Cq* cut-off of 35 cycles (Table 1). None of the non-target samples amplified within this range. Therefore, the analytical as well as the diagnostic specificity of the assay were 100%.

**Table 1:** **Samples used for specificity testing of the BMSB assay**.

**Species**	**Origin**	**Collection date**	**Number of samples**	**Samples tested positive**	**Median *Cq***
*Acrosternum hilare*	Canada	2013	5	0	–
*Banasa dimidiata*	Canada	2013	5	0	–
*Brochymena quadripustulata*	Canada	2013	5	0	–
*Chlorochroa persimilis*	Canada	2013	5	0	–
*Dolycoris baccarum*	Canada	2013	3	0	–
*Erthesina fullo*	Japan	2012, 2013	2	0	–
*Euchistus tristigmus*	Canada	2013	5	0	–
*Glaucias amyoti*	New Zealand	2015	1	0	–
*Halyomorpha halys*	USA, China, Japan	2012, 2013	13	13	19.35
*Nezara sp*.	Japan	2013	1	0	–
*Nezara viridula*	Canada	2013	5	0	–
*Parabrochymena arborea*	Canada	2013	5	0	–
*Podisus maculiventris*	Canada	2013	5	0	–
*Rhaphigaster nebulosa*	Canada	2013	3	0	–

### Analytical sensitivity and performance of the assay

**Figure 1:**
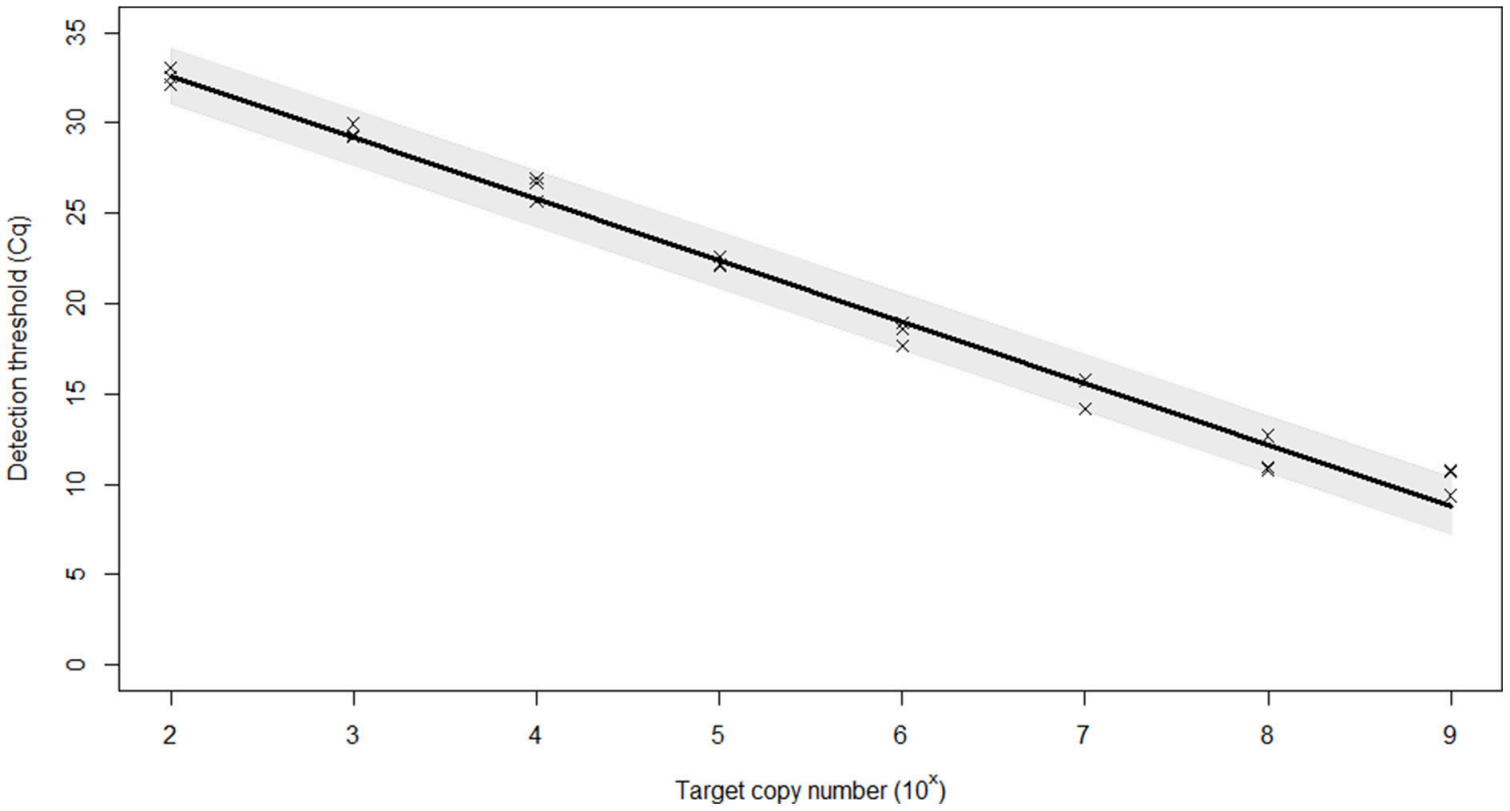
**Efficiency of the real-time PCR assay for the identification of BMSB**. Plasmid dilution series were used to create calibration curves for efficiency calculations. The standard curve built from *Cq* (threshold cycle) values against the log copy number (range = 10^9^–10^2^ copies). The 98% confidence interval of the slope is shaded. Based on curve statistics, the assay efficiency is 97.0% and the *r*^2^ (fit) = 0.99.

The PCR efficiency was calculated at 97.0%, which is within the 95–105% range generally accepted for an efficient PCR reaction (Figure 1). The linear dynamic range of the assay was established as 10^9^–100 copies of template DNA. The correlation coefficient, *r*^2^, of the calibration curve was 0.99. The 98% confidence limits of the linear dynamic range are plotted in Figure 1. Very low variation (%CV) was observed for each of the samples tested within individual runs (repeatability) and between different runs (reproducibility) indicating high assay reproducibility (Table 2).

**Table 2:** **Repeatability and reproducibility of BMSB assay, measured as percentage coefficient of variation (%CV)**.

**Sample**	**Repeatability (assay 1)**	**Repeatability (assay 2)**	**Reproducibility**
Sample 7	1.81	0.49	4.26
Sample 8	1.13	6.73	5.31
Sample 12	3.91	2.99	3.49
Sample 13	0.93	1.22	3.05
Sample 20	0.69	2.05	3.22

*Repeatability is defined as the intra-run variation of a sample and reproducibility as the inter-run variation of a sample. Sample identifiers reflect sample number in blind panel testing (Table 3)*.

### Blind panel validation of the assay

Ten out of the 24 samples of unknown identity were accurately identified as BMSB (Table 3). No false-positive and false-negative results were obtained. All test results matched to the original sample idenitites independently.

**Table 3:** **Blind panel testing of BMSB assay**.

**Sample**	**Life stage**	**Morphological ID**	**Sample origin**	***Cq* (18S control)**	***Cq* (BMSB assay)**	**Diagnostic result**
1	Nymph	*Monteithiella humeralis*	NZ	29.3	–	✕
2	Adult	*Cuspicona simplex*	NZ	19.9	–	✕
3	Adult	*Halyomorpha halys*	USA	17.4	19.4	✓
4	Nymph	*Nezara viridula*	NZ	28.1	–	✕
5	Adult	*Halyomorpha halys*	USA	15.2	20.1	✓
6	Adult	*Erthesina fullo*	Japan	24.5	–	✕
7	Adult	*Halyomorpha halys*	USA	25.0	23.5	✓
8	Adult	*Halyomorpha halys*	USA	19.9	20.5	✓
9	Nymph	*Nezara viridula*	NZ	30.1	–	✕
10	Adult	*Halyomorpha halys*	USA	19.6	22.6	✓
11	Adult	*Erthesina fullo*	Korea or China	18.3	–	✕
12	Adult	*Halyomorpha halys*	USA	18.3	23.1	✓
13	Adult	*Halyomorpha halys*	–	17.2	19.3	✓
14	Adult	*Halyomorpha halys*	Japan	31.2	29.2	✓
15	Egg	*Murgantia histrionica*	USA	25.5	–	✕
16	Adult	*Erthesina fullo*	China	13.0	–	✕
17	3rd instar	*Bagrada hilaris*	USA	18.9	–	✕
18	Adult	*Eysarcoris* sp.	South Korea	21.2	–	✕
19	Adult	*Dictyctus caenoeus*	AK	28.9	–	✕
20	Adult	*Halyomorpha halys*	USA	20.8	18.5	✓
21	Adult	*Halyomorpha halys*	USA	17.9	21.6	✓
22	Adult	*Nezara viridula*	Canada	17.0	–	✕
23	Adult	*Nezara viridula*	Canada	16.8	–	✕
24	Adult	*Nezara viridula*	Canada	16.9	–	✕

*Twenty four samples were provided, of which 10 were BMSB (highlighted). Reactions were performed by an operator without knowledge of each samples identity. All samples were amplified using the TaqMan Ribosomal 18S RNA control assay. The Cq value for each reaction was the only criteria for the identification as BMSB*.

## Discussion

Real-time PCR-based technique have revolutionized pathogen identification throughout the world. Fungi, bacteria and viruses provide excellent examples of plant pathogens that are regularly identified using real-time PCR (Mackay et al., 2002; Bilodeau et al., 2007; Osman et al., 2007; Monti et al., 2013). However, species-specific real-time PCR assays are only available for a fraction of insects, which form a large and diverse group of plant pests. These assays are available for difficult to identify and economically significant pests such as *Thrips palmi* (Walsh et al., 2005) and *Bactrocera latifrons* (Yu et al., 2004). Each species causes severe agricultural damage and are often intercepted as immatures, thereby hindering morphological identification. Similarly, BMSB, which is an emerging pest of high economic and public significance, is also difficult to identify as an egg or in its immature stages. In this study, we have developed a novel real-time PCR assay for the detection and identification of BMSB. Using real-time PCR eliminates post-PCR processing, reducing identification time by several hours. TaqMan probes provide high specificity (Mao et al., 2007) and can be performed at any laboratory.

During the development stage, thorough *in silico* testing was followed by experimental analysis during the specificity testing stage. We analyzed a total of 12 Pentatomidae species during the specificity testing stage (Table 1). In New Zealand, this assay is routinely used for the identification of BMSB, especially from eggs and nymphs. In other countries, we would recommend further specificity testing prior to deployment. One closely related species, with a COI sequence similar to that of BMSB, is *Halyomorpha picus*. Although not tested specifically here, this insect contains multiple mismatches in both primers (F: 2–4 and R: 2–3 sites) and probe (P: 1–5 sites) and is unlikely to successfully anneal to these molecules during the PCR process (Supplemental Figure S1).

One hundred percent diagnostic and analytical specificity, from samples derived from legs of adults, was reported for this assay. Such accuracy and reliability is optimal for diagnostics. The high efficiency and sensitivity, and the ability to identify as low as 100 copies of the target DNA, which is 5 orders of magnitude less than that obtained from a typical extraction further accredits the consistency of the assay results (data not shown). Two well replicated calibration curves validated the limit of detection. Additionally, the extremely low intra-assay and inter-assay variance permits the applicability of the assay across different laboratories a feasible option.

The application of a diagnostic assay within the New Zealand quarantine framework is characterized by three main tenets: high specificity, high sensitivity and swift results. This assay fulfills each of these criteria. The final validation using a blind panel provides further confidence that this assay can be reliably used in a routine diagnostic framework in New Zealand and overseas. It is important to note that border-intercepted organisms were included in the panel to simulate a realistic situation, and border intercepted BMSB samples were also included.

In conclusion, the novel TaqMan qPCR assay developed here is ideally suited for research and diagnostic agencies, in the facilitation of trade, as well as an aid to the border security agencies worldwide, to slow the further spread of this pest. As a rapid, accurate and specific alternative to morphology- or conventional PCR-based techniques, it saves precious quarantine time. With the availability of 96-well or 384-well format PCR machines, which can be coupled with semi-automated extraction methods, this method is amenable to high throughput applications. Such applications are often deemed necessary during large scale surveys for delimiting infestations during an incursion. Like any other diagnostic assay, pre-deployment testing in locations other than New Zealand is highly recommended, to ensure that no false positives exist, although they would be highly unlikely.

## Author contributions

MKD, DL designed the study. MKD, MD, DW performed the experiments. MKD, DW did the analysis. MKD, DW, DL, DA sourced the materials. MKD, DW wrote the manuscript. All authors edited and read the final version of the manuscript.

### Conflict of interest statement

The authors declare that the research was conducted in the absence of any commercial or financial relationships that could be construed as a potential conflict of interest.

## Supplementary material

Supplemental Figure S1 **Neighbor-joining tree of Pentatomidae target sites**. Sequences obtained represent the BMSB and other Pentatomidae sequences used in assay development and validation, as well as BMSB and *H. picus* sequences obtained from online sources. Sequences were aligned and regions corresponding to the forward primer, probe, and reverse primer binding site extracted and concatenated into a single sequence. Tree building was performed using neighbor-joining with a Jukes-Cantor distance model. Support of nodes was obtained through 10,000 bootstrap iterations.

Table S1 **Accession numbers of the sequences of the samples used in the design phase and the specificity-testing phase of the assay development**.
