# Supplementary figures and images for: Real-Time PCR Assay for the Identification of the Brown Marmorated Stink Bug (Halyomorpha halys)
Source: Front Mol Biosci. 2016 Feb 26;3:5. doi: 10.3389/fmolb.2016.00005 (PMC4767901; doi:10.3389/fmolb.2016.00005)

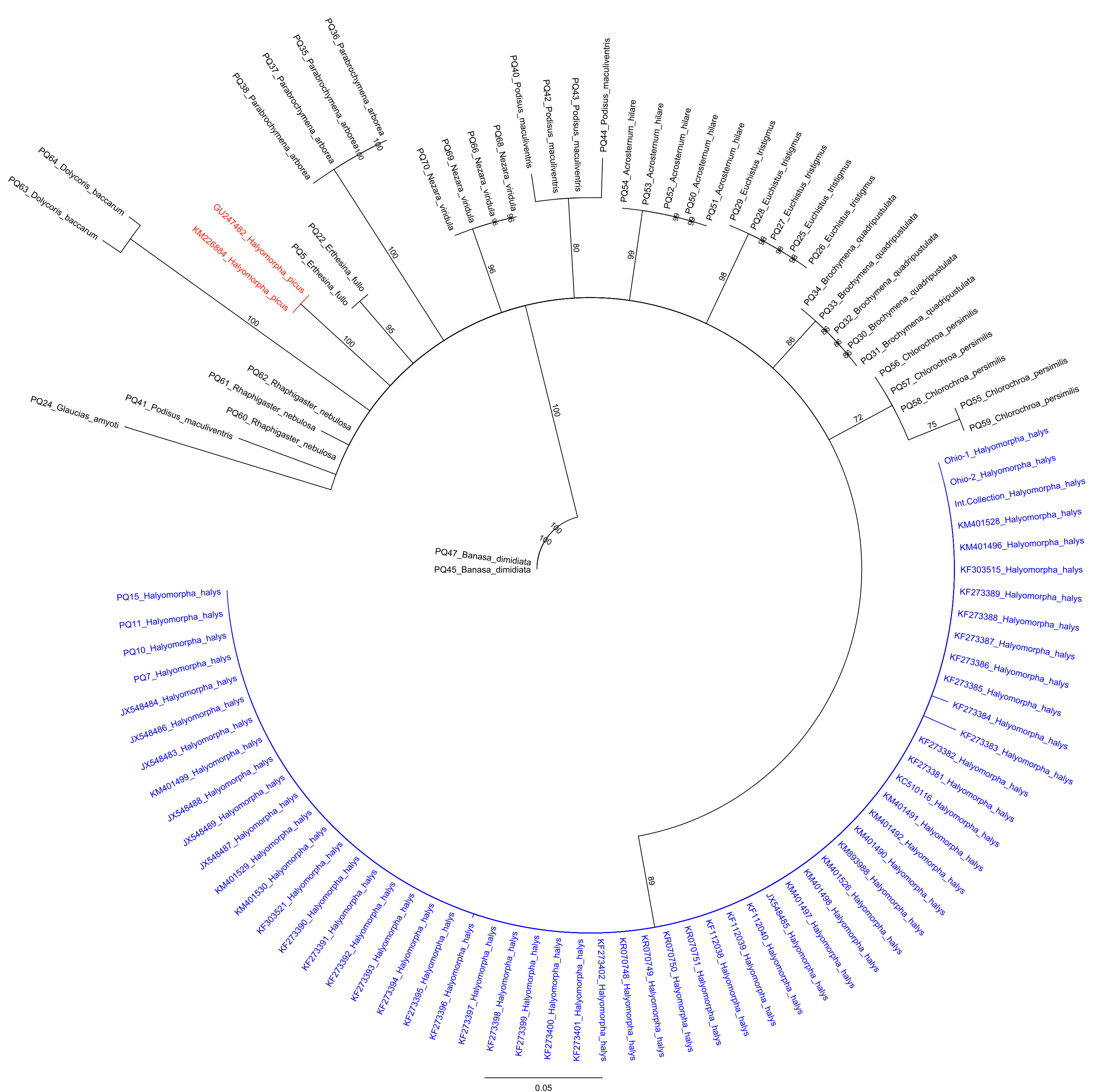

Supplement: Supplemental Figure S1 — Neighbor-joining tree of Pentatomidae target sites. Sequences obtained represent the BMSB and other Pentatomidae sequences used in assay development and validation, as well as BMSB and H. picus sequences obtained from online sources. Sequences were aligned and regions corresponding to the forward primer, probe, and reverse primer binding site extracted and concatenated into a single sequence. Tree building was performed using neighbor-joining with a Jukes-Cantor distance model. Support of nodes was obtained through 10,000 bootstrap iterations. [file Image1.PDF]
